# Supplementary material for: Overexpression of ERAP2N in Human Trophoblast Cells Promotes Cell Death
Source: Int J Mol Sci. 2021 Aug 10;22(16):8585. doi: 10.3390/ijms22168585 (PMC8395336; doi:10.3390/ijms22168585)
Supplement: Supplementary file 1 [file ijms-22-08585-s001.zip › Manuscript Supplemental Tables/Supplemental Table 2 (only).pdf]

Promoting Cell Survival	Promoting Cell Death
COL9A3 ⇩	GPI ⇩
TPI1 ⇩	PGAM1 ⇩
SLC9A3R1 ⇩	HK2 ⇩
TLR3 ⇩	LDHA ⇩
TAPBP ⇩	PKM ⇩
CDC42 ⇧	ALDOC ⇩
ENO3 ⇧	PGM1 ⇩
THBS3 ⇧	PFKP ⇩
SEC31B ⇧	EGLN1 ⇩
	MAML3 ⇩
	EGLN3 ⇩
	TFRC ⇩
	STAT3 ⇩
	PFKFB4 ⇩
	AKR1B1 ⇩
	PDIA3 ⇩
	HSPA5 ⇩
	WFS1 ⇩
	RRBP1 ⇩
	CKAP4 ⇩
	DDOST ⇩
	PDIA4 ⇩
	HSP90B1 ⇩
	OS9 ⇩
	P4HB ⇩
	HSPA1B ⇩
	RFX5 ⇩
	HLA-C ⇩
	CTSS ⇩
	CTSB ⇩
	SLC16A3 ⇩
	TGFB1 ⇩
	ITGB5 ⇩
	FZD7 ⇩
	WNT7A ⇩
	ITPR2 ⇩
	WNT11 ⇩
	SDC1 ⇩
	ITGA5 ⇩
	EZR ⇩
	ENO1 ⇩
	ITGB6 ⇩
	HEY1 ⇩

	ATP6V0A4 ⇩
	EGLN2 ⇧
	PPP1R12B ⇧
