# Supplementary material for: Overexpression of ERAP2N in Human Trophoblast Cells Promotes Cell Death
Source: Int J Mol Sci. 2021 Aug 10;22(16):8585. doi: 10.3390/ijms22168585 (PMC8395336; doi:10.3390/ijms22168585)
Supplement: Supplementary file 1 [file ijms-22-08585-s001.zip › Manuscript Supplemental Tables/Supplementary Table 1 (only).pdf]

Term	*P-Value*	*Adjusted P-value*	*Genes*
Glycolysis / Gluconeogenesis	6.66e-8	1.75e-5	GPI; TPI1; PGAM1; ENO1; ***ENO3***; HK2; ***LDHA***; PKM; PGK1; ALDOC; ALDOA; PGM1; PFKP
HIF-1 signaling pathway	3.30e-5	3.27e-3	***EGLN1***; ***LDHA***; EGLN3; ***EGLN2***; TFRC; STAT3; PGK1; SLC2A1; ENO1; ALDOA; ***ENO3***; HK2
Fructose and mannose metabolism	3.73e-5	3.27e-3	PFKFB4; TPI1; AKR1B1; ALDOC; ALDOA; HK2; PFKP
Protein processing in endoplasmic reticulum	9.74e-5	6.41e-3	ERO1A; PDIA3; HSPA5; WFS1; RRBP1; CKAP4; DDOST; PDIA4; HSP90B1; OS9; CALR; P4HB; SEC24D; ***SEC31B***; HSPA1B
Galactose metabolism	2.32e-4	1.09e-2	GALE; GAA; AKR1B1; HK2; PGM1; PFKP
Lysosome	2.48e-4	1.09e-2	ASAH1; LAMP1; GM2A; NPC1; GAA; SLC17A5; AP1S1; ACP5; ATP6V0A4; CTSD; CTSS; CTSB
Antigen processing and presentation	3.85e-4	1.45e-2	PDIA3; HSPA5; RFX5; ***HLA-C***; CALR; CTSS; HSPA1B; CTSB; TAPBP
Alanine, aspartate and glutamate metabolism	4.64e-4	1.52e-2	GPT2; ADSSL1; ASNS; ABAT; RIMKLA; RIMKLB
Central carbon metabolism in cancer	5.68e-4	1.66e-2	***LDHA***; PKM; PGAM1; IDH1; SLC2A1; SLC16A3; HK2; PFKP
Pentose phosphate pathway	1.59e-3	4.18e-2	GPI; ALDOC; ALDOA; PGM1; PFKP
Steroid biosynthesis	1.93e-3	4.61e-2	SQLE; CYP27B1; DHCR24; DHCR7
Starch and sucrose metabolism	3.64e-3	7.98e-2	GPI; GBE1; GAA; HK2; PGM1
Proteoglycans in cancer	6.05e-3	1.23e-1	TGFB1; ***ITGB5***; FZD7; STAT3; ***FN1***; ***WNT7A***; ITPR2; ***CDC42***; WNT11; SDC1; ITGA5; EZR; ***PPP1R12B***
ECM-receptor interaction	9.79e-3	1.84e-1	***ITGB5***; ***FN1***; SDC1; COL9A3; ITGB6; ITGA5; ***THBS3***
Citrate cycle (TCA cycle)	1.06e-2	1.86e-1	IDH1; IDH2; SUCLG2; ACO1
Phagosome	1.35e-2	2.21e-1	TFRC; LAMP1; ***ITGB5***; ***HLA-C***; CYBA; ATP6V0A4; CALR; ITGA5; CTSS; ***THBS3***
Renal cell carcinoma	1.50e-2	2.32e-1	***CDC42***; EGLN1; EGLN3; TGFB1; ***EGLN2***; SLC2A1
Human papillomavirus infection	1.68e-2	2.45e-1	***ITGB5***; FZD7; ***FN1***; ***HLA-C***; ***WNT7A***; ***THBS3***; ***CDC42***; SLC9A3R1; PKM; WNT11; HEY1; ATP6V0A4; COL9A3; MAML3; ITGB6; ITGA5; TLR3
Thyroid hormone synthesis	2.05e-2	2.84e-1	PAX8; HSPA5; ITPR2; CGA; PDIA4; HSP90B1
